# Psychiatric disorders in psychosocially burdened mothers with young children: a population-based cohort study in Germany

**DOI:** 10.3389/fpsyt.2025.1477336

**Published:** 2025-02-20

**Authors:** Julia Fricke, Marie Bolster, Katja Icke, Natalja Lisewski, Lars Kuchinke, Christiane Ludwig-Körner, Franziska Schlensog-Schuster, Thomas Reinhold, Anne Berghöfer, Stephanie Roll, Thomas Keil

**Affiliations:** ^1^ Institute of Social Medicine, Epidemiology and Health Economics, Charité – Universitätsmedizin Berlin, Corporate Member of Freie Universität Berlin and Humboldt-Universität zu Berlin, Berlin, Germany; ^2^ Unit for Municipal Health Strategies for the City of Freiburg and the District of Breisgau-Hochschwarzwald, Freiburg, Germany; ^3^ Institute of General Practice and Family Medicine, Charité – Universitätsmedizin Berlin, Corporate Member of Freie Universität Berlin and Humboldt-Universität zu Berlin, Berlin, Germany; ^4^ International Psychoanalytic University, Berlin, Germany; ^5^ Department of Child Psychiatry and Psychotherapy for Inpatient and Day-Care Treatments, University of Bern, University Hospital of Child and Adolescent Psychiatry and Psychotherapy, Bern, Switzerland; ^6^ Institute for Complementary and Integrative Medicine, University Hospital Zurich, University of Zurich, Zurich, Switzerland; ^7^ Institute of Clinical Epidemiology and Biometry, University of Würzburg, Würzburg, Germany; ^8^ State Institute of Health, Bavarian Health and Food Safety Authority, Bad Kissingen, Germany

**Keywords:** postpartal, psychiatric disorder, M.I.N.I.-7, population-based study, risk factor

## Abstract

**Introduction:**

Mothers are exposed to a variety of stressors in the early years of their children’s lives, being at risk for mental illness. The aim of our analysis was to estimate the type and frequency of and potential risk factors for psychiatric disorders in mothers with children aged up to three years.

**Methods:**

Based on random population samples from three urban areas in Germany, mothers of infants were recruited for a population-based cohort study as part of the SKKIPPI project. The subjects underwent a two-stage screening process at baseline: A standardized psychiatric diagnostic interview using the Mini International Neuropsychiatric Interview (M.I.N.I- 7) was conducted only with mothers who showed an elevated psychosocial and mental health burden. Mothers with specific psychiatric disorders were invited for follow-up after six months.

**Results:**

814 mothers participated in the psychiatric interview, 304 in the follow-up. At baseline interview, 5% of the mothers had at least one current psychiatric disorder. Generalized anxiety disorders (2%) and major depressive episodes (1%) were the most common disorders. Of these mothers, 42% were still affected at the 6-month follow-up. Risk factors were having at least one strong stressor in life, a severe negative experience in the own childhood, a previously diagnosed psychiatric disorder, a low/medium educational level, and having already received support through early childhood support programs.

**Discussion:**

The occurrence of psychiatric disorders in mothers with young children seemed lower than previously reported, in the majority symptoms disappeared after 6 months. The study provides important information on the frequency of psychiatric disorders in this group and enables care services to be adapted to meet their needs.

## 1 Introduction

Mothers are often exposed to numerous stressors after the birth of their child. Postpartum psychosocial stress can lead to maternal mental disorders, and can also affect the child, which often manifests in regulatory disorders. Regulatory disorders and maternal mental disorders are discussed to disrupt or prevent the formation of a sustainable mother-child bond and to endanger the healthy development of the child. This might have long-term effects on the child’s future emotional wellbeing and child development up to a transgenerational transmission in the case of later parenthood (1–4). This is of considerable relevance as prevalence estimates of mental disorders in the postpartum period are high: up to 20% of the mothers suffer from postpartum anxiety and from postpartum depression (5–7). Of the children younger than 3 years 20% show regulation disorders such as excessive crying, sleep disturbances and feeding problems (8, 9).

Prevalence estimates are often based on patient cohorts or cross-sectional studies that used self-reported instruments and are commonly limited to depression and/or anxiety disorder (5, 6). Population-based observational studies that recruit participants in the general population and cover a broader range of psychiatric disorders assessed by diagnostic interviews are rare. Also, it is unclear as to what extend the postpartum mental symptoms persist or disappear with the stabilization of the psychosocial situation and the somatic health status of the mother. Especially, comprehensive data for the German population are lacking and the psychosocial and mental health care insufficiently addresses the needs of the young families (10).

Against this background, the German SKKIPPI project (Evaluation der Eltern-**S**äugling-**K**lein**KI**nd-**P**sychotherapie mittels **P**rävalenz- und **I**nterventionsstudien/Evaluation of parent-infant psychotherapy using prevalence and intervention studies) was developed, which collected extensive data on the mental health of mothers, risk factors and utilization behavior as part of a cohort study (11–13). In addition, the project tested a psychotherapeutic intervention to strengthen the mother-child bond in mentally ill mothers and children in two RCTs (10, 14, 15). Here we will report results of the cohort study. The aims of the cohort study were to estimate the type and frequency of psychiatric disorders in mothers of young children during their first three years of life and to assess potential risk factors for psychiatric disorders in a German population-based sample.

## 2 Methods

### 2.1 Study design

SKKIPPI was initiated to a) assess the occurrence of psychosocial stress and mental health disorders of parents and their infants in the first years of life in a population based cohort study (11–13) and b) examine the efficacy of a short parent-infant-psychotherapeutic intervention in two randomized controlled trials (10, 14, 15). Based on the results, the psychosocial care of young parents in Germany is to be further developed and improved. The SKKIPPI cohort study is based on a two-step screening design targeting mothers with children aged up to one year in three German cities (Berlin, Leipzig and Flensburg). Ethical approval for this study was obtained from the ethics committee of the Charité - Universitätsmedizin Berlin (EA2/201/18). The study was registered in the German Clinical Trial Registry on February 8th 2019 (DRKS-ID: DRKS00016653).

### 2.2 Sample

A random sample of 30,000 children aged <12 months was drawn from the registry offices in three German cities (Berlin and Leipzig in East, Flensburg in North Germany). Invitation letters to participate in the study were addressed to the mother while both parents were invited to complete an online questionnaire using an individual QR code. Inclusion criteria were: biological or adoptive mother or father of the child up to the age of 12 months at the time of study sampling, minimum age of both parents 18 years old, registered as residents in Berlin, Leipzig, or Flensburg, written consent, and sufficient language skills to fill out the questionnaire that was available in German, English, Turkish, or Arabic language. As the (successive) mailing of the invitations to participate in the study, including reminders in the event of non-response, to the addresses drawn, took time, some of the infants were already older than 12 months when the mother answered the first questionnaire.

### 2.3 Data collection and assessments

The data collection consisted of three steps:

First step: screening for participants with an elevated mental health risk and to collect relevant characteristics as determinants (online screening questionnaire).

Second step: assessing more in-depth information on psychiatric disorders in mothers at risk of a mental health or psychosocial burden and their children, as well as detailed data on kind and frequency of health and social care utilization (telephone interview at baseline).

Follow up (FU): re-assessing all participants of the baseline interview with certain predefined psychiatric disorders after 6 months (FU telephone interview).

Data were collected between March 2019 and July 2020 for the first screening step, between June 2019 and October 2020 for the second screening step of the baseline assessment (in depth psychiatric interview) and January 2020 and April 2021 for the follow-up assessment.

The online questionnaire was developed as a first screening step by the SKKIPPI cohort study team and included 40 questions on sociodemographic characteristics, perinatal stressors and characteristics, individual parental stressors, current and life time mental health problems in the parent including possible own childhood trauma, and regulation disorders of the child (12). Mothers who appeared to have an elevated mental health or psychosocial burden based on the online questionnaire (first step), were invited to participate in a clinical diagnostic interview as a second screening step. Having an elevated mental health risk was based on a predefined criterium developed by experts of the SKKIPPI cohort study team. The study team used a scoring system with points ranging from 0 to 5 for each of the 40 questions and a cut-off of 10 points indicating an elevated mental health risk (12). The subsequent clinical interview was conducted via phone by trained study staff to identify mental health diagnoses using the Mini International Neuropsychiatric Interview (M.I.N.I. 7) (16). The M.I.N.I. is a validated structured interview developed to diagnose the 17 most common mental health disorders according to DSM-V and ICD-10. We adapted the M.I.N.I.-7 in German and English to use via phone and excluded modules that were not suitable for diagnoses over the phone (suicidality & psychotic disorder). The very small number of non-native German or English mothers were able to choose between the German or English version of the M.I.N.I. for their interview. The interview further identified the presence of depressive symptoms during the postpartum period using additionally the Edinburgh Postnatal Depression Scale (EPDS), indicators of parental burnout using the Parental Burnout Assessment (PBA), regulatory problems in the child using the Questionnaire for Crying, Feeding and Sleeping, as well as health care and social service utilization (self-developed questionnaire) (16–19). Mothers with a psychiatric disorder according to the M.I.N.I. (at least one current diagnosis), the EPDS (score of ≥ 13) or the PBA (score of ≥ 76) were invited to participate in a follow-up interview six months later using the same instruments. A detailed description of the SKKIPPI cohort study design has been published elsewhere (13).

Overall, 5,946 parents (4,984 mothers and 962 fathers) participated in the first screening step of the SKKIPPI study. Of the 4,984 mothers, 1,185 (23.8%) were considered to have an elevated mental health risk based on the first screening step and were invited to participate in the in depth psychiatric interview. The average time between first screening and psychiatric interview was 2.3 months (SD=2.2). Results of the online questionnaire have in detail been reported elsewhere (12).

For the analysis presented in this manuscript we included all mothers who participated in the second screening step of the SKKIPPI cohort study at baseline and completed at least one module in the M.I.N.I. telephone interview. Mothers who dropped out after the first screening step and did not receive the M.I.N.I. interview were excluded from this analysis.

### 2.4 Outcomes

The primary endpoint of this analysis was the occurrence (frequency) of at least one current psychiatric diagnosis at baseline and at the time of follow-up after 6 months.

Secondary outcomes were the occurrence of the individual diagnoses: major depressive episode (past two weeks), manic and hypomanic episodes (current), panic disorder (past month), agoraphobia (current), social anxiety disorder (social phobia) (past month), obsessive-compulsive disorder (past month), posttraumatic stress disorder (past month), alcohol use disorder (past 12 months), substance use disorder (non-alcohol) (past 12 months), anorexia nervosa (past three months), bulimia nervosa (past three months), binge-eating disorder (past three months), or generalized anxiety disorders (past 6 months) (all assessed by M.I.N.I. interview at the time of baseline screening step 2 and follow-up). In addition, the change of occurrence between baseline screening step 2 and follow-up was assessed.

As potential risk factors the following items were included into the analysis: age of the mother, German language skills, educational level (ISCED), single parenthood, recipient of state payments, support through early childhood programs, childcare burden due to chronic illness, severe negative experiences in own childhood, previous diagnosed mental health disorder, unplanned pregnancy, pregnancy complications, study participation during the SARS-CoV-2 pandemic, and having at least strong/very strong stressors (**Supplementary Table 1**). This information was taken from the mother’s self-report in the online questionnaire of the first screening stage (12). Mothers could rate stressors in their relationship, job, lack of social support or other conflicts on a 4-point scale (not at all/a little/rather more/strongly or very strongly).

### 2.5 Statistical analysis

Results are presented as absolute and relative frequencies for categorical data with two-sided 95% confidence intervals (CI). Occurrences of psychiatric disorders in the second screening step of the baseline assessment were analyzed in relation to 1) the entire SKKIPPI cohort of mothers, who participated in the first screening step and 2) in relation to those participating in the second screening step. Mothers who qualified for the second screening step but did not participate were excluded from the analyses.

Associations between risk factors and the primary endpoint were analyzed using multiple logistic regression yielding odds ratios (OR) with 95%-CI. Missing values were not replaced. All results are interpreted exploratively. All calculations were carried out in R and R Studio version 2021.09.0 (20) with the involvement of a statistician (S.R.).

Sensitivity analysis: As data collection coincided with the SARS-CoV-2 pandemic and was performed during pandemic related containment measures, the occurrence of mental health disorders was compared between those who completed the interview before and during the SARS-CoV-2 pandemic for both the second screening step and the follow-up assessment. The date of March 16th, 2020, when large scale containment measures including the closing of schools were implemented in Germany, was used as the date to distinguish between pre-pandemic participation and pandemic participation. The occurrence of at least one psychiatric disorder was compared between the mothers who completed the M.I.N.I. interview prior to March 16th 2020 (pre-SARS-CoV-2) and after (during SARS-CoV-2).

## 3 Results

**Figure 1:**
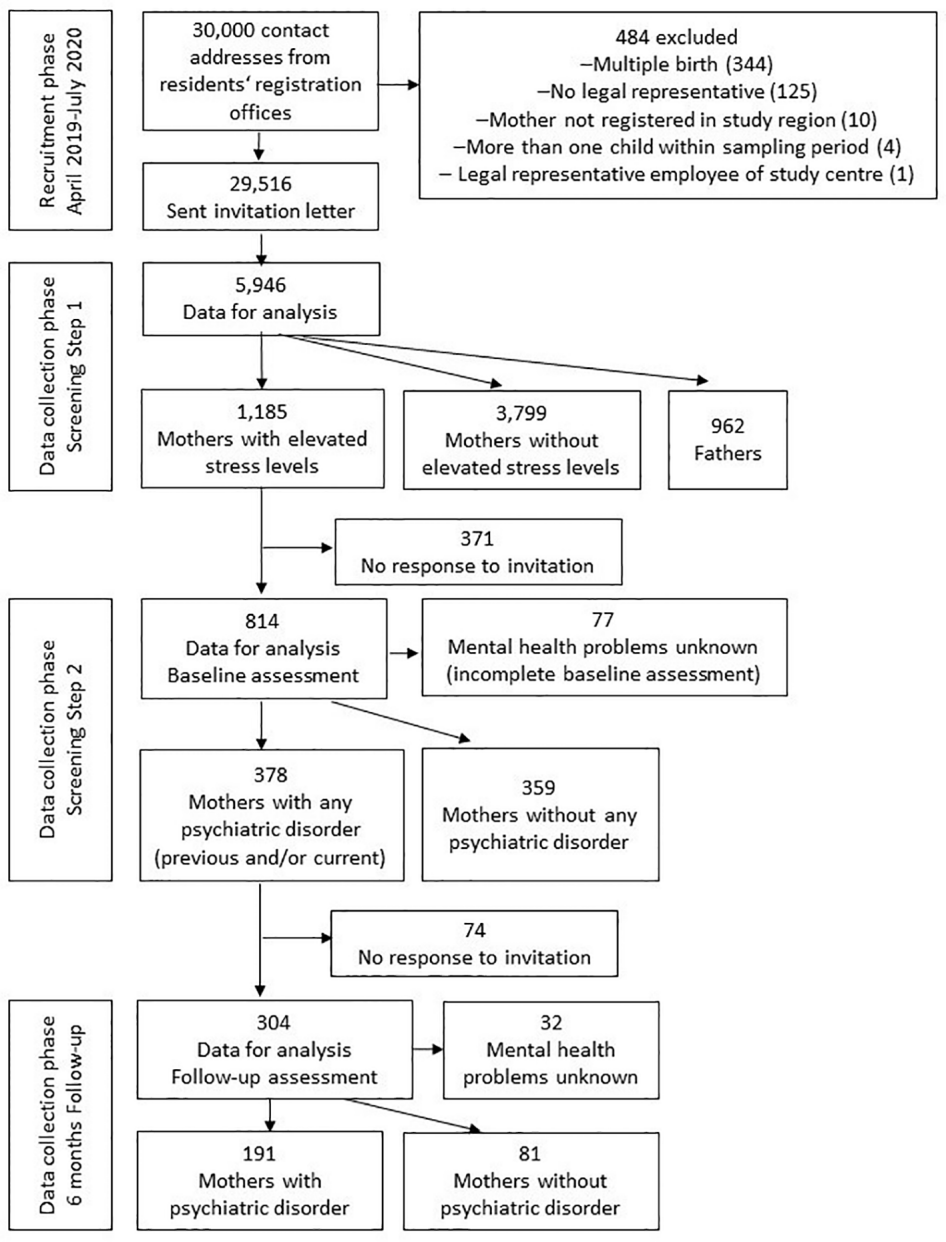
Flowchart of the SKKIPPI cohort study recruitment and participation.

In total, 814 mothers participated in the psychiatric interview and 304 in the follow-up assessment, 371 were invited but did not respond to invitation, 74 did not respond to the invitation to follow-up assessment after 6 months (**Figure 1**). The average time between psychiatric interview and follow-up was 6.8 months (SD=1.3).

Most participating mothers lived in Berlin, had a high level of education, and were between 30 and 39 years old (**Table 1**). The average age of the child was 18.0 (SD=3.4) months during the second screening step and 24.8 (SD=3.6) months during follow-up.

**Table 1:** Baseline characteristics of 814 mothers participating in the second screening step of the SKKIPPI cohort study baseline assessment.

	Mothers, n (%)
**Study site**	
- Berlin	675 (82.9)
- Flensburg	12 (1.5)
- Leipzig	109 (13.4)
- Other region #	18 (2.2)
**Mean age, years (SD) 34.4 (4.8)** **Age group**	–
- ≤29	117 (14.4)
- 30-39	588 (72.2)
- 40-49	108 (13.3)
- ≥50	1 (0.1)
**Country of birth**	
- Germany	675 (82.9)
- Other country	139 (17.1)
**Native language**	
- German	679 (83.4)
German language skills (if not native German, self- assessment)	135
- Very good/good	113 (83.7)
- Medium	11 (8.1)
- Bad	11 (8.1)
**Educational level**	
- Low (ISCED 1)	5 (0.6)
- Middle (ISCED 2)	120 (14.7)
- High (ISCED 3)	679 (83.4)
- Unknown	10 (1.2)
**Currently in a partnership**	739 (90.8)
**Single parent**	96 (11.8)
Number of children* < 18 years in the household	
1	448 (55.1)
2	277 (34.0)
3 or more	89 (10.9)
**Receipt of state payments (n=1 missing)**	158 (19.4)
**Support through early intervention programs**	249 (30.6)

#Mothers who moved between date of address selection by public authority and study inclusion or who do not live at the registration address; *Including index child, that is, the child that led to the study inclusion; ISCED, International Standard Classification of Education.

**Figure 2:**
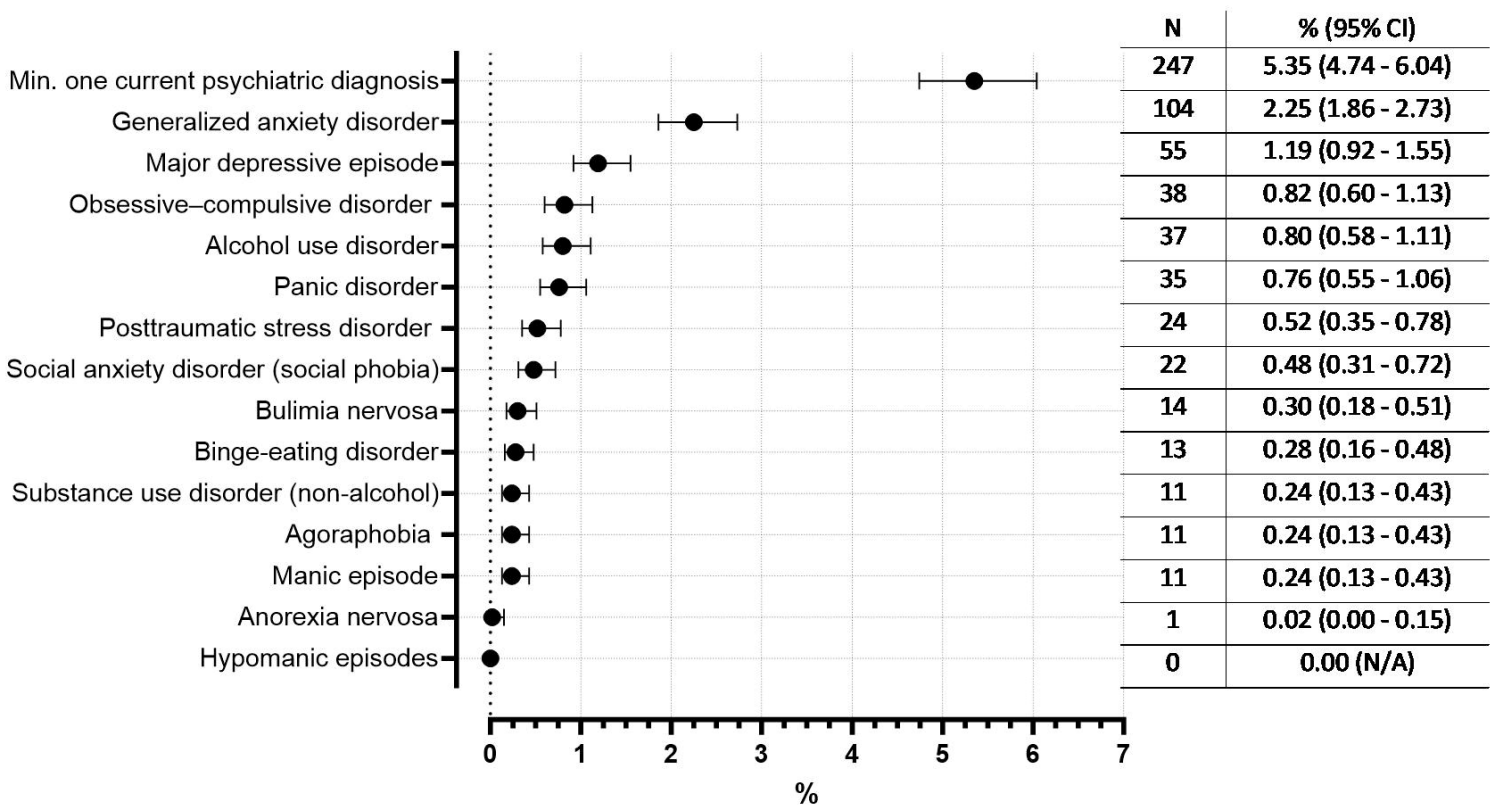
Occurrence of current psychiatric disorders based on M.I.N.I. and prevalence estimates for the general population of postpartum mothers (% with 95% CI, as a proportion of all 4,613 mothers with clear mental health status in the SKKIPPI study population). Excluded are mothers with a positive first screening in the online questionnaire but missing second screening step and thus unclear mental health status (n=371).

**Figure 3:**
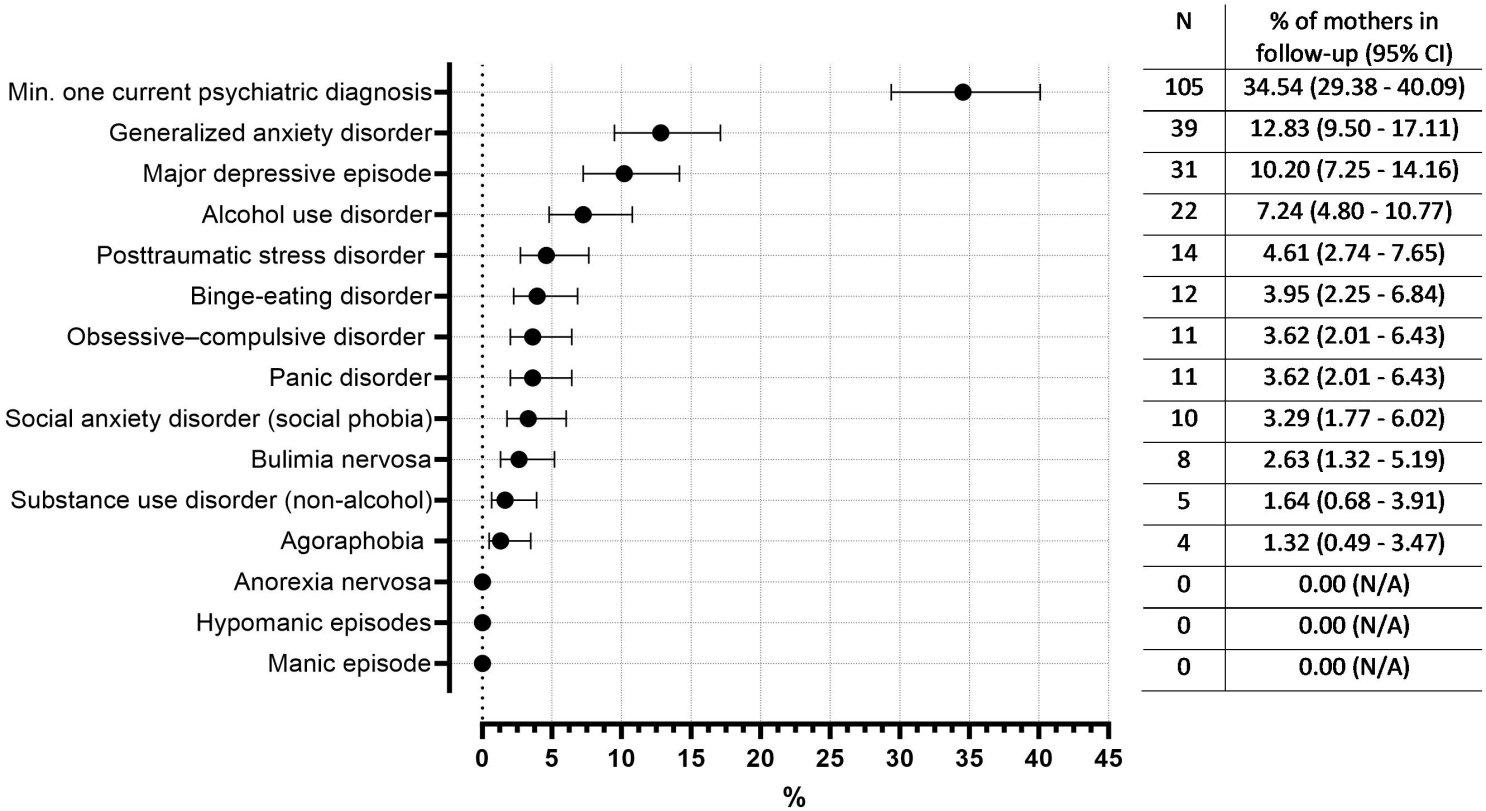
Occurrence of current psychiatric disorders at follow-up (in % with 95% CI, as a proportion of all mothers in the follow-up (n= 304).

Approximately 5.4% of the 4,613 mothers with data on their mental health status had at least one current psychiatric diagnosis (**Figures 2**, **3**). The most common current disorders were generalized anxiety disorder (2.3% of all participating mothers), major depressive episode (1.2% of all participating mothers) and obsessive-compulsive disorder (0.8% of all participating mothers) (for proportions of disorders among those who participated in the screening step 2 see **Supplementary Figure 1**).

In the follow-up assessment including only mothers who had a psychiatric disorder in the second screening step of baseline assessment and responded to follow-up invitation, approximately 34.5% of mothers had at least one current psychiatric diagnosis. The most common disorders in the follow-up were generalized anxiety disorder (12.8%), major depressive episode (10.2%) and alcohol use disorder (7.2%) (**Figure 3**).

**Figure 4:**
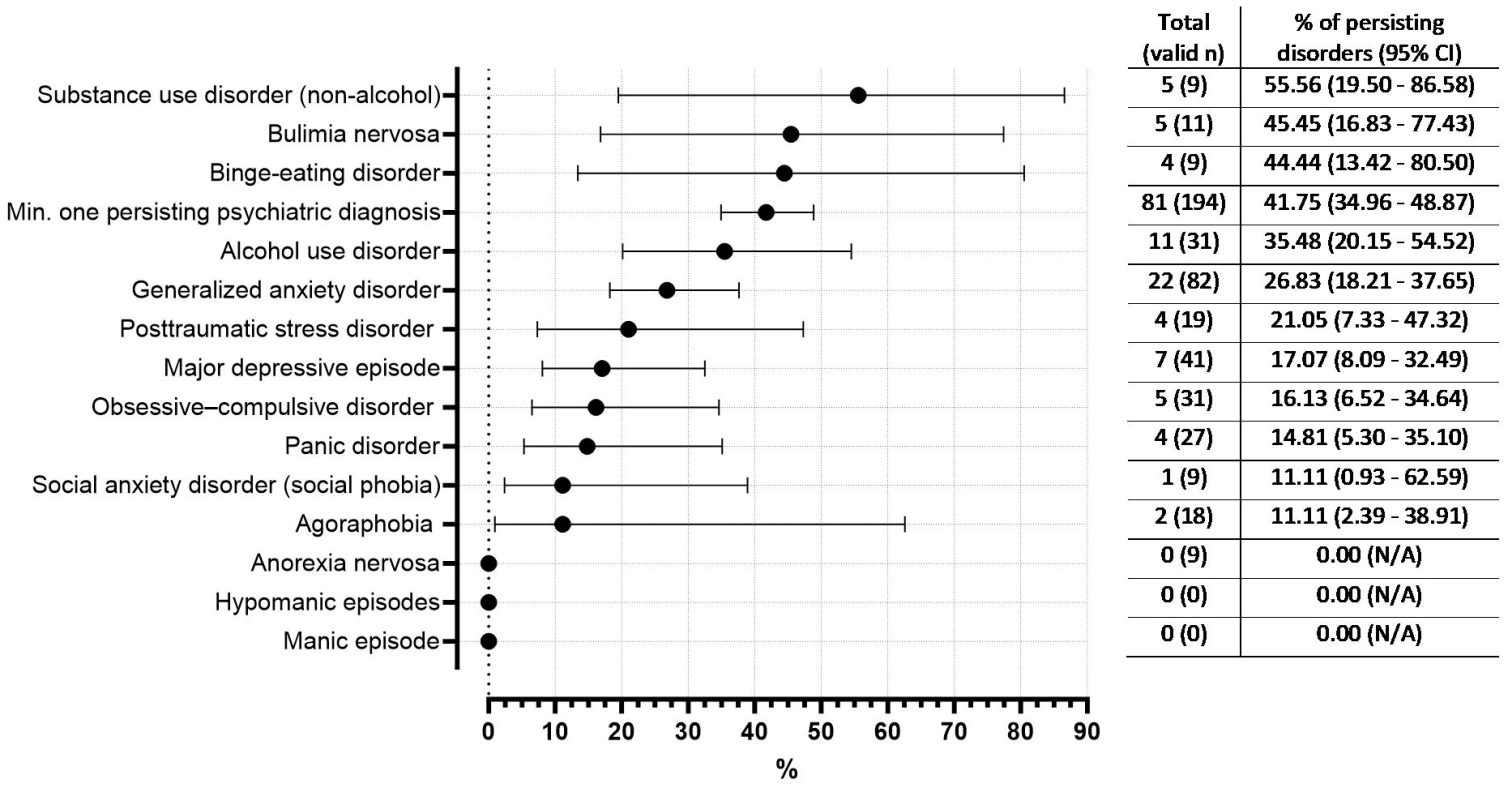
Occurrence of psychiatric disorders persisting between psychiatric assessments at baseline and follow-up (in % with 95% CI, n= 304 participants in follow up). Valid n refers to those mothers who had the specific psychiatric diagnosis in the baseline assessment and were not dropouts in the follow-up. Therefore, the valid n per denominator differs per diagnosis.

Of those mothers with at least one psychiatric diagnosis in the second screening step, 41.8% continued to have a diagnosis six months later in the follow-up. The most common disorders that persisted until follow-up were substance use disorder (55.6%), bulimia nervosa (45.5%) and binge-eating disorder (44.4%) (**Figure 4**).

In the second screening step of the baseline assessment, 69% of participants completed the interview before start of the SARS-CoV-2 pandemic. In the follow-up only 8% participated before the pandemic. A sensitivity analysis of M.I.N.I. interviews during the pandemic has shown, that participants during the pandemic had higher occurrences of at least one current psychiatric diagnosis, major depressive episode, panic disorder, agoraphobia, social anxiety disorder, obsessive-compulsive disorder and bulimia nervosa compared to the group that was interviewed before the pandemic. However, generalized anxiety disorder was lower in the pandemic group, compared to the pre-pandemic group. All other disorders remained relatively similar in both groups (**Supplementary Figure 2**).

To identify risk factors for having at least one mental health diagnosis, the data of 4,527 mothers were included in the regression model, 86 were excluded due to missing values. The strongest predictors for a psychiatric diagnosis were having at least one strong/very strong stressor in life, having a severe negative experience in the own childhood, having had a previously diagnosed mental health disorder, having participated in the study during the SARS CoV-2 pandemic, receiving support through early childhood support programs, and having a low/medium/other educational level according to the ISCED classification (**Table 2**).

**Table 2:** Factors associated with outcome of at least one psychiatric diagnosis (multivariable logistic regression).

Outcome: min. one psychiatric diagnosis (n=4,527)		
Risk factor	Odds Ratio	95% CI
Age (mother) in years	0.96	(0.93-1.00)
German language skills (good/very good/native)^1^	0.83	(0.39-2.05)
Educational level (ISCED) (low/medium/other)^2^	**1.76**	**(1.21-2.53)**
Single parent	1.07	(0.60-1.83)
Recipient of state payments	1.17	(0.77-1.77)
Support through early childhood programs	**1.92**	**(1.36-2.70)**
Child care burden	1.25	(0.79-1.94)
Severe negative experience in own childhood	**4.04**	**(2.99-5.47)**
Previous diagnosed mental health disorder	**3.98**	**(2.93-5.42)**
Unplanned pregnancy	1.39	(0.98-1.95)
Pregnancy complications	1.34	(0.98-1.82)
Study participation during SARS-CoV-2 pandemic	**2.61**	**(1.85-3.66)**
Min. one strong/very strong stressor ^3^	**5.01**	**(3.69-6.81)**

Data of all mothers without missing values were included into the analysis.

^1^Reference group: poor/very poor German language skills.

^2^Reference group: high educational level.

^3^According to the mother’s self-report in the online questionnaire used in the first screening step. Stressors in the partnership, job, lack of social support or other conflicts could be rated as “not at all/a little/rather more/strongly or very strongly”.

Bold values represent risk factors with statistical significance.

## 4 Discussion

### 4.1 Main findings

In our population-based cohort study in three German cities approximately 5% of the mothers had at least one current psychiatric diagnosis according to the M.I.N.I. interview. Psychiatric disorders that were recorded most frequently were generalized anxiety disorders and major depressive episodes. More than 40% of mothers who had a diagnosis at the baseline assessment also had a diagnosis at the 6-month follow-up interview. Various predictors for having a mental health disorder could have been identified like reporting a strong/very strong life stressor, having had a previously diagnosed mental health disorder or severe negative experiences in the mother’s own childhood.

### 4.2 Comparison with other studies

Studies examining mental health disorders after birth usually assess the prevalence of individual disorders and focus primarily on depressive and anxiety disorders. Meta-analyses on postpartum depression and anxiety disorders in the postpartum period revealed numbers that vary widely around the world. They reported prevalence rates between 4% and 64% for depressive symptoms, 15% for anxiety symptoms, 10% for anxiety disorders and 6% for generalized anxiety disorder. These rates are mostly based on self-reporting questionnaires like the Edinburgh Postnatal Depression Scale (5, 6). The occurrences for these psychiatric disorders assessed by the M.I.N.I. interviews in the SKKIPPI cohort study are lower than the above-named numbers and also lower than those assessed in other German studies with prevalence rates between 3% and 6% for depressive and around 11% for anxiety disorders (21–23). One reason for this difference could be that the prevalence rates in the meta-analysis are often overestimating prevalence rates since they are predominantly based on self-reporting questionnaires and not on psychiatric diagnostic interviews (5, 6). Another reason for this difference could be that observation periods vary between the studies. For example, the other German studies examined the time period immediately after birth compared to the SKKIPPI cohort study which examined the period related to the first three years of life after the birth of the child. On the other hand, in an Australian cohort study, which assessed depressive symptoms during the first four years after birth, point prevalence ranged from 8 to 14.5% with the highest prevalence rate recorded at the 4-year follow up (24). This study aligns with previous studies that find fluctuating trajectories of psychiatric symptoms in mothers. In a sample of nearly 5,000 mothers assessing symptoms at four different time points in the Upstate KIDS-study, Putnik et al. identified different trajectories of depressive symptoms within the 36 months postpartum. They showed that only around 5% of the mothers with depressive symptoms had high depressive symptoms at all assessment points and around 75% low depressive symptoms at all assessment points (25). However, the Upstate KIDS-study used the 5-item-EPDS for diagnosis which differs from the M.I.N.I. in the diagnostic threshold. It is conceivable that a selection bias can also explain the differences. Mothers who do not feel impaired or burdened, as well as mothers who are seriously ill, may not be likely to take part in a study like ours, which requires a significant time investment from the participants. Another study by Jacques et al. also found different trajectories with the first two years of life with 4% of the mothers showing high depressive symptoms at all assessment points (26). In the SKKIPPI cohort sample 17% of the mothers who had a diagnosis of depression in the baseline M.I.N.I. assessment still had depressive symptoms at the 6-months follow-up interview. This means that depressive symptoms in more than 80% of the mothers with depressive symptoms at the baseline interview remitted or disappeared, meaning they received treatment, or the symptoms have remitted spontaneously.

Longitudinal data of anxiety symptoms in the first years after birth are less frequently assessed than depressive symptoms. In a Canadian cohort study between 15% and 17% of the mothers reported anxiety symptoms in the first three years after birth, but data were assessed using a self-reporting instrument (27). In addition, diagnostic criteria are more severe for anxiety disorders than anxiety symptoms, which could partially explain the differences in occurrences to the SKKIPPI cohort study.

In addition to depressive episodes and generalized anxiety disorders, occurrences of all other psychiatric complaints were low in the SKKIPPI cohort study with less than 1% for each psychiatric disorder. A meta-analysis of studies using structured diagnostic interviews, reported a prevalence of 2.4% for postpartum obsessive-compulsive disorder (28). Comparable data for other mental disorders in the postpartum period are rare or lacking at all.

Looking at the various predictors for psychiatric disorders assessed in the SKKIPPI cohort study, the most pronounced - reporting a life stressor, having had a previously diagnosed mental health disorder or severe negative experiences in the mother’s own childhood – are well-known determinants for postpartum psychiatric disorders. For example, severe negative experiences during childhood have been shown to be a risk factor for pre- and postpartum depression (29, 30). Life stressors which has been assessed in our study comprised for example lack of social support, a known risk factor that has been reported in many studies on postpartum depression (1, 31). The same applies for a previous diagnosis of a mental health disorder (1, 31). Study participation during SARS-CoV-2 pandemic turned as well out to be a strong predictor. We interpret this to mean that this predictor is a proxy for stress caused by external (social, economic, other) crises that affect individuals’ everyday lives.

The educational level of the participants was very high. This is a common finding in epidemiologic studies and reflects a general underrepresentation of people with medium and low levels of education (32, 33). We tried to reach more people with a lower level of education by developing a questionnaire that was as short and simple as possible and by making several languages available. Future studies should therefore try to apply specifically adapted strategies, such as outreach surveys to specific clientele or, for example, more targeted sampling. The results are therefore not easily transferable to the population as a whole. It can be assumed that people with a medium or low level of education have fewer resources to cope with psychosocial problems and that the frequency of mental health symptoms may be higher.

### 4.3 Strengths and limitations

A major strength of the SKKKIPPI study is its population-based design using random samples from several German registry offices. In Germany, previous studies in this area are often based on outpatient- or hospital-based data sets (22, 23). Another strength is the implementation of a structured psychiatric diagnostic interview instead of self-reporting questionnaires like the Edinburgh Postnatal Depression Scale. The M.I.N.I. interview covers many different psychiatric disorders, not just depressive or anxiety disorders, which are usually the focus of studies in the postpartum period. Information was also collected on the frequency of obsessive-compulsive disorders and alcohol abuse. To our knowledge, this is the first study in Germany which assessed mental health disorders in the first years after birth using the M.I.N.I. interview. Another strength of the study is that it also allows conclusions to be drawn about the time course of postpartum disorders, as a significant proportion of the mothers took part in the follow-up assessment after 6 months.

The study has also several limitations. Firstly, psychiatric diagnostic interviews were only done with mothers considered at risk of a mental health or psychosocial burden as determined by the first step online questionnaire. The SKKIPPI cohort study may have missed mothers having a psychiatric disorder showing low scores on this online questionnaire. However, since the questionnaire includes the assessment of many different risk factors for psychiatric disorders, we estimate the probability to be rather low. Based on this argument, the calculated frequencies must be considered conservative estimates. Secondly, another limitation is potential self-selection, whereby mothers who are less burdened are more likely to participate in the study. The interviews were only conducted in German or English, leaving mothers unable to complete the interview in either of these languages underrepresented. Specifically in urban regions persons without sufficient knowledge of the language are often socially deprived and hesitate to follow invitations. Additionally, most of the mothers had a high level of education, which means that mothers with a low level of education and the associated risk of mental illness are also underrepresented. Thirdly, the samples sizes from the population registration offices all come from urban areas, so no conclusions can be drawn about the transferability of the results to the rural population with more traditional family constellations. Fourthly, the psychiatric diagnostic interviews were conducted only with mothers, although fathers were also invited to complete the first step online questionnaire. The underlying assumption was that mothers would be more interested and could invest more time to participate in this more time intensive part of the study. Future studies should implement psychiatric diagnostic interviews also with affected fathers to get a more comprehensive picture of the situation of all parents in Germany. Another analysis of the data from the SKKIPPI cohort study showed different patterns of risk factors for mental disorders for mothers and fathers (34). In depth psychiatric interviews with fathers would also allow individual triggering and contributing factors in the families to be examined in detail. However, this would have gone beyond the scope of this study, which focused primarily on the aspect of population representativeness and prevalence. It would also be important to target specifically same-sex parents additionally to complete this picture. Fifthly, there was a significant attrition of participants between first interview and follow-up, which is why the cases for the individual diagnostic groups in the analysis were sometimes very small and the confidence intervals large. The validity of the follow-up results is therefore considerably lower. Sixthly, the SKKIPPI cohort study did not collect any follow-up data on possible psychiatric treatment of the mothers before inclusion in the study or until follow-up. In this respect, we cannot make any statement about the extent to which the changes in the mothers’ mental state are due to therapeutic interventions or spontaneous remission. Finally, we decided to exclude the complete assessment of the diagnosis suicidality and psychotic disorder due to the difficult nature of asking questions about these disorders via phone. To get some information about these two mental health disorders, we asked some basic questions, but did not analyze the data because it was not diagnostically interpretable. However, it is possible that the omission of these two diagnostic modules underestimated the frequency of psychiatric disorders in the study sample.

## 5 Conclusion

Data on maternal mental health in the first years after birth are mostly based on self-reporting instruments and are also not available for all mental health conditions. The data collection in the SKKIPPI cohort study used a psychiatric diagnostic interview comprising all kind of psychiatric disorders. The occurrences for the different psychiatric disorders are lower than those reported in the literature. Nevertheless, the affected mothers may need help and support in order not to compromise their own mental health or that of their child. The findings on the occurrence of mental disorders in postpartum mothers suggest that more attention should be paid to mental health in the routine care of this group of individuals. This is primarily a task for gynecologists and also general practitioners and can be achieved through the use of established short screening instruments. Pediatricians, who regularly see mothers as part of their check-ups, should also develop an awareness of mental disorders in mothers. Future studies should collect longitudinal data with several follow-up examinations, in order to better understand the course of the various psychiatric diseases in the first years of life after birth and thus to be able that affected parents get targeted treatment and support available.

## Data Availability

The raw data supporting the conclusions of this article will be made available by the authors, without undue reservation.
