# Supplementary material for: Psychiatric disorders in psychosocially burdened mothers with young children: a population-based cohort study in Germany
Source: Front Psychiatry. 2025 Feb 20;16:1477336. doi: 10.3389/fpsyt.2025.1477336 (PMC11883137; doi:10.3389/fpsyt.2025.1477336)
Supplement: Supplementary file 1 [file Supplementaryfile1.docx]

Supplementary Material

Supplementary table 1: Regression model covariates

| Outcome: min. one mental health disorder according to the MINI questionnaire | |
| --- | --- |
| Covariates/ risk factors | **Variable type** |
| Age of the mother | Numeric/continuous (in years) |
| German language skills | Binary (good/very good/native vs. medium/bad) |
| Educational Level (ISCED) | Binary (high vs. medium/low other) |
| Single Parent | Binary (yes/no) |
| Recipient of state payments | Binary (yes/no) |
| Support through early childhood programmes | Binary (yes/no) |
| Childcare burden | Binary (yes/no)  At least one of the following burdens:  -Min. 1 child with a disability or chronic illness in the household  - min 3 children under 6 years in the household  - multiple birth |
| Severe negative experience in own childhood | Binary (yes/no) |
| Previously diagnosed mental health disorder (lifetime) | Binary (yes/no)  Includes Depression, Anxiety, OCD and psychosis |
| Unplanned pregnancy | Binary (Yes/no) |
| Pregnancy complications | Binary (yes/no) |
| Study participation during COVID pandemic | Binary (before/during COVID pandemic)  Participation in the study before or after March 16^th^ 2020 (start of pandemic containment measures, including school closures, in Germany) |
| Min. one strong/very strong stressor | Binary (yes/no)  Min. one of the following stressors (relationship problems, work problems, lack of social support, financial worries or a chronic disease) * |

*According to the mother’s self report in the online questionnaire used in the first screening step. Stressors in the partnership, job, lack of social support or other conflicts could be rated as “not at all/a little/rather more/strongly or very strongly”


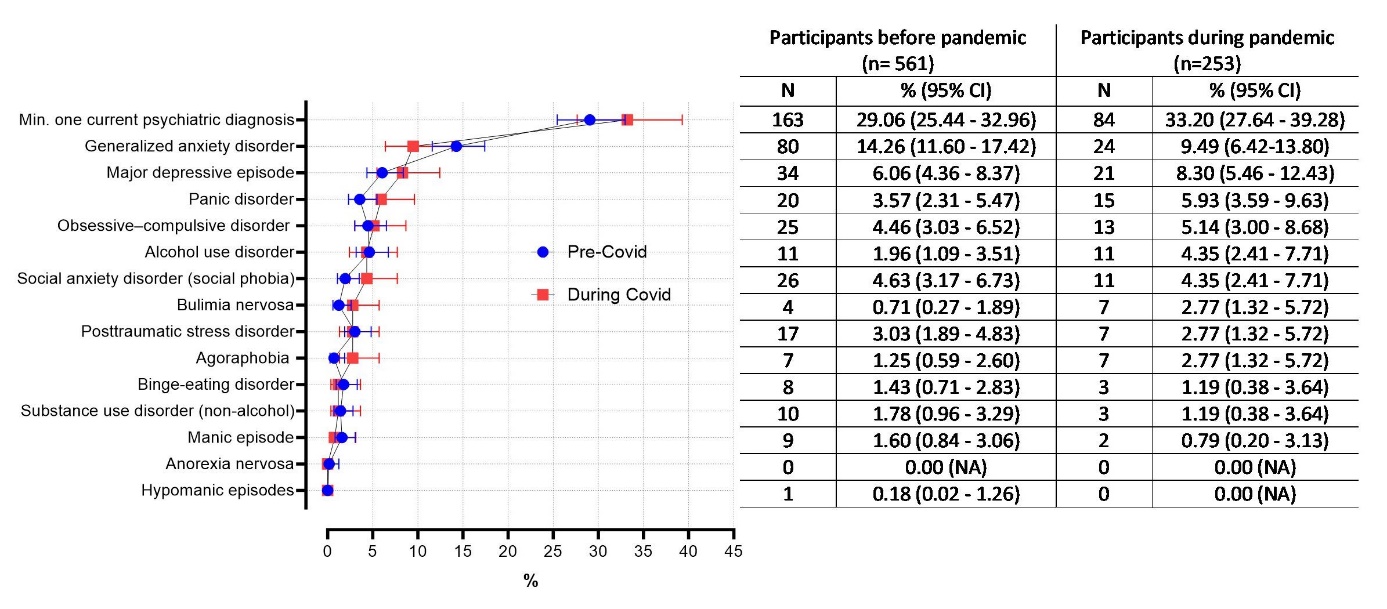


Supplementary figure 1: Occurrence of current psychiatric disorders (in % with 95% CI, as a proportion of all mothers who participated in the second screening assessment (n= 814).


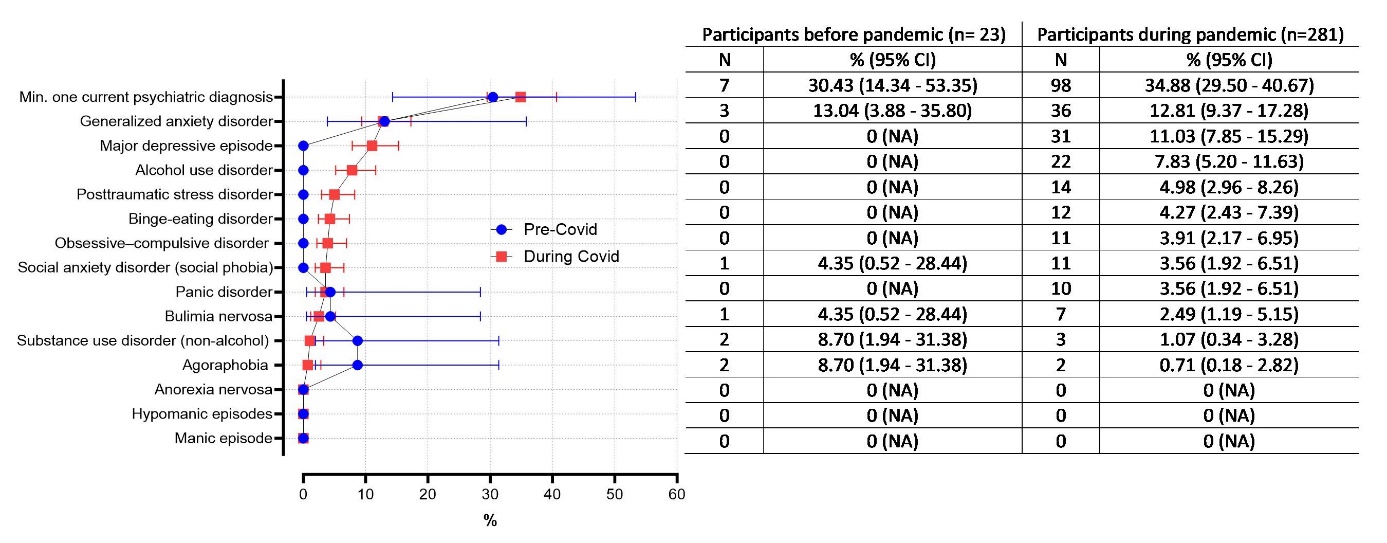
 Supplementary figure 2: Occurrence of current psychiatric disorders according to the M.I.N.I., comparison between group of mothers with baseline assessment before SARS-CoV-2 pandemic (n= 561) and group of mothers with baseline assessment during SARS-CoV-2 pandemic (n=253) (in % with 95% CI).
